# What constitutes ‘good practice’ in early intervention for psychosis? Analysis of clinical guidelines

**DOI:** 10.1111/camh.12229

**Published:** 2017-08-08

**Authors:** Paolo Corsico, Michelle Griffin‐Doyle, Ilina Singh

**Affiliations:** ^1^ Centre for Social Ethics and Policy School of Law The University of Manchester Manchester UK; ^2^ Department of Psychiatry University of Oxford Warneford Hospital Oxford UK; ^3^ Department of Psychiatry and Wellcome Centre for Ethics and Humanities University of Oxford Oxford UK

**Keywords:** Clinical guidelines, early intervention, ethics, good practice, psychosis, adolescence

## Abstract

**Background:**

Early Intervention in Psychosis (EIP) services have been implemented with the dual aims of preventing harmful outcomes associated with early‐onset psychosis and improving prognosis. However, concerns have been raised regarding the ethical implications of involving young people in EIP services. One way to ensure high ethical standards and promote good practice in EIP delivery is through governance of clinical practice. This study aimed to investigate the normative dimensions of good practice in EIP through examination of clinical guideline documents published in England over the past 15 years.

**Methods:**

A total of 14 clinical guidelines and relevant policy documents for EIP were retrieved and analysed using a mixed inductive and deductive thematic approach. Themes were derived from the data itself, whereas the development of broader categories was performed through a constant comparison with the scientific literature describing ethical issues in EIP.

**Results:**

Ethical touchpoints of good practice in EIP included both procedural and substantive factors, which were seen to be interdependent and mutually constitutive. These ethical touchpoints were largely implicit in the documents analysed. Procedural requirements of EIP service delivery consisted of norms and rules pertaining to EIP service structure, adherence to codes of ethics, inclusivity, patient and family centredness and appropriate treatment provision. Substantive factors consisted of moral attributes that should be cultivated by healthcare professionals working in EIP: competency, empathy, sensitivity and trustworthiness.

**Conclusions:**

We argue that, to ensure good practice in EIP, procedural and substantive ethical expectations embedded in EIP guideline documents should be made explicit in EIP service and care delivery. We suggest that the procedural and substantive factors highlighted in this paper contribute useful dimensions for the eventual evaluation of good practice in EIP services across England.

## Abstract 2

Read the Commentary on this article at doi: 10.1111/camh.12265

**Key Practitioner Message**

- Ethical touchpoints of good practice in early intervention for psychosis go beyond the mere provision of sound professional guidance.
- An important distinction exists between procedural and substantive ethical expectations embedded in clinical guideline documents; such as ethical requirements of service delivery (procedural) and moral attributes required of healthcare professionals (substantive).
- Procedural and substantive ethical dimensions should be explicitly considered in early intervention design and delivery.
- The ethical dimensions highlighted in this study should be considered as key variables in the evaluation of good practice in early intervention services across England.

## Introduction

In the last twenty years, Early Intervention in Psychosis (EIP) services have been introduced worldwide (Bird et al., 2010). EIP services implement early detection and intervention for young people who experience a First Episode of Psychosis (FEP), or those who are in the putative prodromal phase of psychosis – usually defined as At‐Risk‐Mental‐State (ARMS), High Risk (HR) or Ultra High Risk (UHR) for psychosis (Fusar‐Poli, Yung, McGorry, & van Os, 2014; Yung & Nelson, 2011).

Early Intervention in Psychosis services were implemented in the United Kingdom in 2001, following the publication of the Mental Health Policy Implementation Guide (MHPIG) by the Department of Health (2001). In England, guidance for EIP is provided by the Initiative to Reduce the Impact of Schizophrenia (IRIS) Network (IRIS Network website) and by the National Institute for Health and Care Excellence (NICE 2016). NICE Guidelines, NICE quality standards and NICE pathways are each designed to inform clinical practice and to provide healthcare professionals with standardised advice regarding implementation of EIP services. Additional scientific and clinical guidance for EIP services is provided in England by the IRIS Network (IRIS, 2012), a professional network which has been active since 1998.

Accumulating evidence since the initiation of early intervention strategies in first‐episode and at‐risk populations suggests that EIP can prevent some of the harmful outcomes associated with early‐onset psychosis and schizophrenia and can improve prognosis (Amminger et al., 2011). However, specific concerns have been raised with regard to the ethical implications of involving young people in EIP services. These concerns include stigma and labelling associated with early diagnosis of mental health problems (Appelbaum, 2015); the potential adverse impacts of intervention on young people's developing sense of personal identity and autonomy over the developmental course (Cassetta & Goghari, 2015); and risk–benefit ratio in the provision of pharmacological treatment (Jorm, 2012; Warner, 2005). The ethical risks of stigma, self‐understanding and early pharmacological treatment are considered particularly acute in relation to the ARMS population, in which putative symptoms do not reach a clinically significant threshold and do not necessarily involve repeated or impairing patterns of behaviour or cognitions (Broome & Fusar‐Poli, 2012). Given the young age of a majority of service users enrolled in EIP, families’ and carers’ vulnerability has also been identified as a sensitive ethical issue in the implementation of early intervention strategies (McCann, Lubman, & Clark, 2011), along with risk communication (Mittal, Dean, Mittal, & Saks, 2015) and concerns about privacy and confidentiality of service users’ and family's information (Cassetta & Goghari, 2015).

One way to ensure high ethical standards and to minimise the potential for harm in EIP is through governance of clinical practice. A key governance mechanism is the clinical guideline. The clinical guidelines for good practice in EIP, developed by IRIS and NICE, can be viewed as professional codes of conduct leading to ‘good practice.’ In medical research as in medical ethics, ‘good practice’ identifies the ethical requirements that characterise the sound conduct of clinical research and of medicine (International Conference on Harmonisation, 1996).

A body of literature in moral philosophy goes further to conceptualise the idea of ‘practice’ in moral terms, arguing that the concept of ‘good’ practice entails a normative dimension (MacIntyre, 2007). For MacIntyre, the value of a practice is predicated on the presence of a trusting relationship in which the goods of that practice are acknowledged, shared and debated (2009). One ground for these relationships is the professional bodies in which, and through which, standards of ‘good practice’ are derived. These standards are important for healthcare professionals and healthcare recipients alike; indeed, according to MacIntyre's account, a clinician who enables patient flourishing should be a flourishing practitioner.

As outlined above, a range of ethical concerns have already been raised with regard to EIP services; however, none of these has yet focused on the normative dimensions of ‘good practice’ in EIP services. In this article, we investigate these dimensions through examination of a body of clinical guideline documents published over the past 15 years. We identify and explicate latent and explicit ethical touchpoints of good practice in EIP, with the aim of specifying the criteria thought to produce excellence in EIP service delivery.

## Methods

### Data collection

Data collection took place between January and March 2016. Clinical guidelines and relevant policy documents for EIP services were initially retrieved through a combination of database‐assisted (Web of Science and Scopus) literature reviews, in depth exploration of the scientific literature on EIP, and Google searches, through combinations of relevant keywords. Keywords used for database searches included: “Early intervention”, “Psychosis”, “Schizophrenia”, “Guideline(s)”, “Guidance” and “Policy(‐ies)”. Only documents published in England were considered for inclusion. Clinical guidelines were found to be published by NICE and IRIS; policy documents were found to be published by the UK Department of Health and NHS England. Documents were retrieved from institutional websites.

### Characteristics of the documents

Materials included comprised: (a) policy documents describing aims and structure of EIP services in England; (b) clinical guidelines for EIP services provided by national institutions in England. The documents included covered a time span of 15 years (2001 to 2016), and had to be published before March 2016 in order to be eligible for inclusion. Of the documents originally retrieved, 14 were eligible for the final analysis as they referred to EIP services in England.^1^ The 14 documents comprise: policy documents from the UK Government Department of Health (*n* = 2); NICE CGs (*n* = 3); NICE Quality Standards (*n* = 1); NICE pathways (*n* = 6); other relevant clinical guidelines for EIP services published in England (*n* = 2). A list of all the documents included and document abbreviations may be seen in Table 1.^2^

**Table 1:**
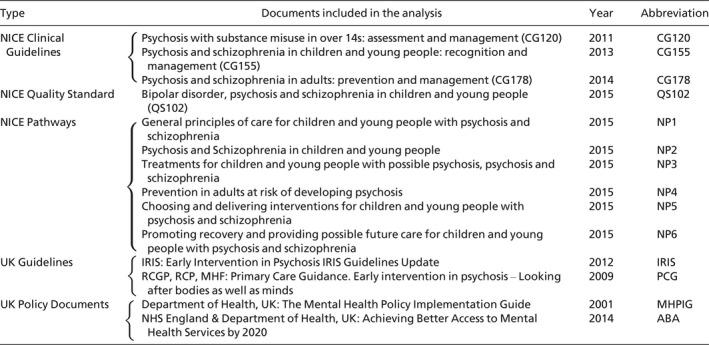
List of documents included in the analysis

### Data analysis

The documents were analysed using a thematic approach as outlined by Braun and Clarke (2006). The process of extracting and constructing themes and categories from the data was performed as a mix of inductive and deductive thematic analysis (Fereday & Muir‐Cochrane, 2006): themes were derived from the data itself, but the development of broader categories was performed through a constant comparison with the scientific literature which describes ethical issues in EIP services delivery.

One author (PC) performed the literature search for clinical guidelines on EIP. The documents were initially read by different team members. A coding structure was developed after the first in depth reading, and was then applied to the documents through a CAQDAS Software (NVivo10). All the documents were coded according to the first coding structure. During the second in depth reading process, themes were developed through a close study of the data by the authors who reached consensus on the final coding frame/thematic map. Themes were grouped in higher order categories, which were organised under two different headings: (a) Ethical requirements of EIP service delivery; and (b) Moral attributes of clinicians. The final coding frame was checked for reliability by different team members and the documents were then recoded accordingly.

## Results

### Policy documents, guidelines and principles of good practice

Two key EIP policy documents have been produced by the UK Department of Health and by NHS England, respectively: the ‘Mental Health Policy Implementation Guide’ (MHPIG) published in 2001; and ‘Achieving Better Access to Mental Health Services by 2020’ (ABA), published in 2014. Both documents emphasise the moral and ethical dimensions of early intervention in mental health, as well as several principles of care that should guide the design and delivery of EIP services in England.

The following MHPIG principles of care inform the provision of mental healthcare in the UK, including early intervention in mental health (MHPIG, p. 4):

1. the centrality of the service users and their families,
2. the focus on service users’ needs and preferences, and
3. the necessity to avoid over diagnosis in the provision of mental health services, which in MHPIG is referred to as the need to avoid “the blight of the ‘not invented here’ syndrome”

MHPIG also implements the 11 principles established by the Mental Health National Service Framework (MHNSF) in 1999, which includes the following (MHPIG, p. 6):

1. avoid discrimination
2. promote independence
3. promote continuity of care, and
4. involve services users and their carers in service design and delivery

MHPIG lists several principles of care that should specifically characterise the provision of EIP services, including (MHPIG, p. 44):

1. culture, age and gender sensitive
2. family oriented
3. meaningful and sustained engagement
4. emphasis on the management of symptoms rather than the diagnosis, and
5. treatment provided in the least restrictive and stigmatising setting

MHPIG does not identify any prodromal or High‐Risk group (Fusar‐Poli et al., 2014) as potential EIP service users, a facet added to EIP services more recently following the debate on the risk‐syndrome included in the DSM‐5 (Broome & Fusar‐Poli, 2012).

The Department of Health and NHS England jointly issued ABA in 2014, claiming a strong moral case for the implementation of better access to mental health services, including EIP. Although mainly focused on budgetary issues related to the improvement of mental health services in England, ABA positions better access to EIP services as a political requirement using ethical language; e.g.:

[Better access to mental health services] is the right thing to do, both morally and ethically (ABA, p. 1)

The clinical guidelines included in the present analysis differ with regard to length, structure and their applicability to EIP services. The length of the documents ranges from two pages (PCG) to 57 pages (CG178). Most documents were not specifically designed to provide professional guidance only for EIP services; rather, they cover the provision of EIP services along with clinical guidance for other services such as Child and Adolescent Mental Health Services (CAMHS) and primary care. Appendix S1 summarises the clinical guidelines that refer to EIP services reviewed for this paper.

### Themes, categories and ethical touchpoints of good practice in EIP

**Figure 1:**
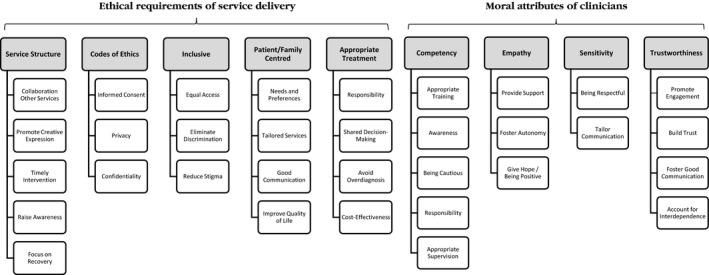
Thematic map and final coding structure

Thirty‐three themes were derived from the analysis and were then grouped in nine high order categories, which represent the ethical touchpoints of good practice in EIP services in England. In other words, the nine high order categories identify the ethical arguments embedded in clinical guidelines for early intervention in psychosis in the English context. The nine high order categories were organised under two different headings: (a) ethical requirements of EIP service delivery and (b) moral attributes of clinicians. The final thematic map may be seen in Figure 1.

Five categories were found to fall into ethical requirements of service delivery: (a) service structure; (b) codes of ethics; (c) inclusive; (d) patient and family centred and (e) appropriate treatment. Four categories were found to fall into moral attributes of clinicians: (a) competency; (b) empathy; (c) sensitivity and (d) trustworthiness. A list of the 33 themes, nine high order categories and two headings, tabulated in function of the themes covered by the documents included in the analysis may be seen in Table 2.

**Table 2:**
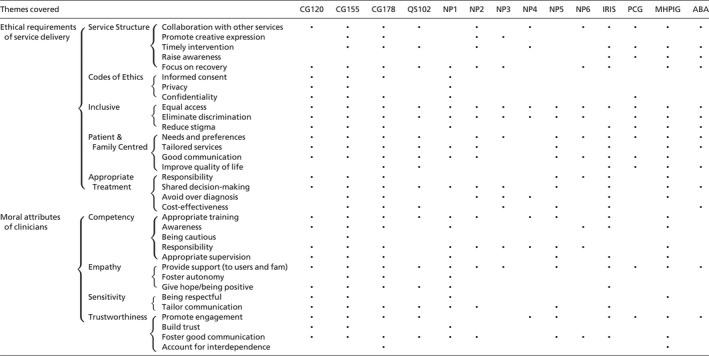
Themes covered by the documents included in the analysis

### Ethical requirements of service delivery

Ethical requirements of EIP service delivery can be described as the ethical norms and codes of conduct that should characterise the structure and delivery of EIP services in order to promote good clinical practice. In this sense, they identify norms, rules and principles of care pertaining to healthcare delivery services, and not directly to virtues or moral attributes that should be cultivated by healthcare professionals. Relevant sample quotes for each theme under the “ethical requirements of service delivery” heading may be seen in Appendix S2.

#### Service structure

The documents analysed indicate that EIP services should be set up in such a way that they can achieve certain valued ends. Collectively, these ends can be summarised as an institutional effort to align EIP services more closely with the nondiagnostic, preventive philosophy widely accepted for physical health. Therefore, one key requirement of EIP service structure is to foster collaboration with primary care services and CAMHS, and to work in partnership with the third sector and voluntary organisations. To avoid the need for diagnostic labels and for drug treatments, raising awareness of EIP and timely intervention are required. A focus on recovery, found in almost all the clinical and policy documents, also prioritises the ends of prevention over diagnosis and treatment, and potentially avoids the harms of medicalisation and pathologisation. Two NICE guidelines (CG155 and CG178) and two NICE pathways (NP2 and NP3) placed emphasis on nonpathologising intervention activities that foster creative expression and confidence, such as art and music. Such interventions are proposed to improve clinical outcomes, and to help “[…] people to express themselves and to organise their experience into a satisfying aesthetic form” (CG178, p. 25).

#### (Adherence to) codes of ethics

Clinical guidelines provided by NICE (CG155 and CG178) underline the importance of ensuring that discussions with service users take place in a setting where privacy and confidentiality are respected. CG155 underlined the importance of being clear with service users, carers and families about the limits of confidentiality when collaborating with other services (p. 13). Specific codes of ethics were not mentioned by the policy documents included in the analysis or most of the clinical documents. Among NICE pathways, only NP1, which is dedicated to the principles of care for children and young people with psychosis and schizophrenia, explicitly referred to codes of ethics.

#### Inclusive

A widely recognised ethical requirement of EIP services is that they should promote equal access, eliminate unlawful discrimination and reduce stigma. According to almost all the documents, service delivery should be youth‐friendly, tailored to the needs of ethnic minorities and to people from diverse cultural backgrounds, and it should promote equality of opportunity. In addition, as stigma associated with early referral to mental health services is recognised as a major ethical issue in the implementation of EIP services (Appelbaum, 2015), the documents underline the importance of providing a low stigma environment within EIP services.

#### Patient & family centred

According to almost all the documents, a patient and family‐centred approach is pivotal to the implementation of EIP services. Services should take into account the needs and preferences of both users and their families and carers, with regard to treatment decision‐making and patient engagement. EIP Services should be tailored to young and heterogeneous populations, and they should be designed in order to promote good communication among healthcare professionals, users and carers on the one hand, and with other services, such as CAMHS and primary care on the other hand. The policy documents CG178, QS102, IRIS and PCG emphasised the importance of improving users’ and carers’ *quality of life*. None of the NICE pathways mentioned the improvement of quality of life as an ethical requirement of service delivery.

#### Appropriate treatment

Appropriate treatment decisions are at the core of clinical guidance for EIP services. The documents maintain a distinction between treatments for those who have experienced a FEP and those who are in the prodromal or ARMS population. Almost all the documents reported the necessity to involve users and carers in the decision‐making process regarding the provision of treatments. At the same time, both NICE (CG120, CG155 and CG178) and IRIS specified that healthcare professionals should maintain responsibility for the final decision regarding the treatments provided to service users. In addition, in line with the suggestions formulated by the Department of Health in MHPIG and ABA, EIP services should try to avoid over diagnosis – a point that is relevant to young people in the prodromal phase of psychosis (Raven, Stuart, & Jureidini, 2012), and they should implement cost‐effective treatment options.

### Moral attributes of clinicians

Moral attributes of clinicians working in EIP services can be described as the moral skills that should be cultivated by healthcare professionals in order to foster good clinical practice. In other words, moral attributes of clinicians can be interpreted as virtues that exemplify the skills and inform the activities of clinicians performing their duties in EIP services. Therefore, the moral attributes are recognised by in the character of the clinician and in her/his practices. Relevant sample quotes for each theme under the “moral attributes for clinicians” heading may be seen in Appendix S3.

#### Competency

In line with the indications of MHPIG, NICE and IRIS guidelines emphasise the importance of ensuring that healthcare professionals working in EIP achieve an exceptional knowledge base in EIP delivery and in the interpersonal dimensions of working with young service users and families from very diverse backgrounds. Clinical experience and awareness, negotiation skills, and responsible delivery of interventions are viewed as integral to the high level of competency required of healthcare professionals working in EIP services; and these skills must be supported by ongoing supervision (CG155, CG178, CG120, IRIS).

#### Empathy

Collectively, the documents describe the provision of empathic, nonjudgmental support to patients and families as essential to early intervention strategy in EIP populations, carers and families, especially with regard to the prodromal or ARMS population. Such support is necessarily attuned to the interests of the patient and to fostering her/his autonomy. Being positive, optimistic and “giving hope” to users and families with regard to recovery was explicitly mentioned by NICE and IRIS guidelines as a core requirement of healthcare professionals working in EIP: “Provide treatment and care in the least restrictive and stigmatising environment possible and in an atmosphere of hope and optimism […]” (CG178, p. 27).

#### Sensitivity

MHPIG, CG120, CG155 and NP1 explicitly mention the requirement of “being respectful” of people's gender, background and socioeconomic factors: “Be respectful of and sensitive to children and young people's gender, sexual orientation, socioeconomic status, age, background (including cultural, ethnic and religious background) and any disability” (CG155, p. 15). The virtue of sensitivity which is required of healthcare professionals working in EIP also includes the necessity to tailor communication to people of different ages and from diverse cultural backgrounds, as well as the prescription of using plain language, avoiding clinical jargon, using communication aids and making sure that service users, carers and families understand what is being said.

#### Trustworthiness

CG120, CG155 and NP1 explicitly note building trust with users and families. Relations of trust are essential to ethical EIP services, and are seen to depend upon clinicians’ moral attributes and practices. MHPIG, NICE and IRIS guidelines include the prescription of fostering good communication among stakeholders, a substantive requirement that provides the conditions for trust (QS102, p. 47). The Department of Health in MHPIG, CG178 explicitly emphasise that the everyday activities of EIP clinicians must take into account the “interdependence’ of the young person, suggesting that trust must be mutually established and shared among stakeholders. Finally, the trustworthy clinician supports one of the most important goals, as outlined in these documents: the promotion of service‐user and carer early engagement with services, no matter the service‐user's mental health status.

## Discussion

Overall, IRIS and NICE guidelines for good clinical practice in EIP are consistent in identifying ethical requirements of service delivery as well as moral attributes of clinicians working in EIP. The two institutions cover a consistent set of themes and provide similar recommendations and clinical guidance in early intervention in psychosis. The frequent use of terms and concepts showing a strong connection with the ethics domain within MHPIG and ABA, along with the recurrence of those themes in the clinical guidelines, illustrates the extent to which ethical touchpoints of good practice influence clinical guidance for healthcare professionals working in early intervention services.

The collective policy and clinical documents reviewed here suggest that good practice in NHS England EIP services is built around a set of core, principled challenges grounded in a preventive model:

1. EIP service users will, ideally, not become patients
2. EIP service users must be engaged in order to prevent over diagnosis or worsening of symptoms
3. Some EIP service users are at higher risk than others, and these will need diagnosis and more intensive treatment

Our findings suggest that to meet these challenges of good practice, EIP services must achieve certain structural characteristics, and its practitioners must have certain moral attributes. The distinction between ethical requirements of service delivery and moral attributes of clinicians is central to the present analysis. Clinical guidelines and policy documents prescribe how EIP services should be structured, in order to achieve the goals of intervening early in treating psychosis and schizophrenia, and promoting the youth‐friendly, low stigma care environment seen to promote recovery and improve quality of life in young EIP service users. At the same time, the clinician–patient relationship is positioned as a key dimension of good practice in EIP, and the healthcare professional must acquire and practice certain moral skills in order to meet the normative challenges inherent in EIP services. In this sense, ethical requirements of service delivery and moral attributes of clinicians working in EIP are interconnected and interdependent, the latter being contingent upon the ethically sound implementation of EIP services.

The distinction between ethical requirements of service delivery and moral attributes of clinicians resembles the one between norms, rules and codes of conduct on the one hand, and character, values and virtues on the other (Beauchamp & Childress, 2009; MacIntyre, 2007). Put another way, our analysis clarifies that, in EIP services *procedural* ethics, entailed in service delivery and structure of EIP, and *substantive* ethics, enacted through the virtuous qualities and behaviours of clinicians, are seen to be mutually constitutive (Øvreeide & Matjan, 2012).

Codes of ethics include common procedural ethical concerns that have been widely addressed within the medical ethics and research ethics debates, at least since the publication of the Belmont Report (Beauchamp & Childress, 2009); e.g. informed consent, assessment of Gillick competence in children and young people (Wheeler, 2006), privacy and confidentiality. Interestingly, explicit mention of mainstream codes of ethics was not evident in IRIS, nor in the majority of the NICE pathways. The lack of explicit mention of such codes might signal a disconnection between the theoretical debates in clinical ethics on the one hand, and practice dimensions of EIP services on the other. As it is, the ethical requirements of EIP service delivery in EIP policy and clinical documents, at this stage, are rarely anchored in generally accepted medical ethics standards. Further research is needed to evaluate whether this disconnection has any impact on practice, and whether it forms a barrier to the delivery of ethical EIP services.

Likewise, it is important to point out that, although service users’ engagement, shared decision‐making and good communication are seen to make up part of the ethical structure of the EIP service, the potential for moral conflicts among the different stakeholders involved in EIP service delivery was not tackled by the documents analysed here. No professional guidance was offered in the case of moral conflicts among service users, carers and/or the EIP clinician. The absence of guidance here is notable, given the potential for strong disagreements and disruptive processes in such a stressful and emotionally laden service context. Moreover, the capacities of EIP service users will vary as a function both of developmental age and of symptoms, making an appropriate response to conflict and disruption all the more complex. A body of qualitative research has started exploring young service‐users’ pathways to early intervention, their understandings of the aims of early intervention, and their experiences of the treatment dimensions of EIP services for young people (Boydell, Stasiulis, Volpe, & Gladstone, 2010; Lavis et al., 2015; Lester et al., 2012). This research appropriately engages EIP service users as active participants in their clinical care, and EIP clinicians might find this literature useful in addressing and managing moral conflicts that involve young service users.

## Conclusions

The present paper provides a review and analysis of the ethical arguments embedded in clinical guidelines for early intervention in psychosis in England. Ethical arguments embedded in clinical guidelines for EIP can be described as the ethical touchpoints of good practice in EIP services. A key finding of this analysis is that the implementation of good practice in the delivery of early intervention for psychosis simultaneously involves ethical requirements of service delivery – which fall into the domain of procedural ethics – and moral attributes that should be cultivated and “practiced” by healthcare professionals – which pertain to the domain of substantive ethics. This duality is implicit across the body of documents we analysed; at no time is it made explicit to practitioners or stakeholders. Therefore, it is unclear whether, and to what extent healthcare professionals working in EIP in England are aware of the procedural and substantive ethical touchpoints of EIP practice. In the absence of explicit communication on these touchpoints, healthcare professionals are likely to refer to internalised guidelines rather than official and explicit guidelines and codes of practice (Gabbay & le May, 2004). In addition, given the recent emphasis placed by the NHS on the inclusion of the ARMS population in the early intervention strategy, and considered the controversy regarding diagnostic thresholds and appropriate intervention in this preclinical population (see Whale, Thompson, & Fraser, 2017), a stronger focus on the procedural and substantive ethical dimensions of EIP may be essential to the ethical implementation of the early intervention strategy in England.

Ideally, procedural and substantive components of the ethical implementation of EIP services should be mutually enabling, mirroring the strong correlation described in the documents analysed here. To embed this coconstitutive approach to good practice into EIP services and service delivery, the specific elements of procedural and substantive ethics identified in this article must first be made explicit in EIP settings. It will then be important to ensure that EIP professionals are enabled to enact both the ethical requirements of service delivery and the moral attributes of clinicians that are at the core of EIP service and care. Eventually, the dimensions of good practice in EIP service structure and care identified in this article can usefully inform the evaluation of good practice in EIP services across England. Lastly, given recent attempts to broaden the scope of early intervention from psychosis to the entire spectrum of youth mental health – IEPA has currently changed its name to IEPA (Early Intervention in Mental Health) – we propose that the present findings be translated to support the ethical introduction of early intervention services to tackle diverse areas of youth mental health.

## Ethical information

No ethical approval was required for this article.

## Supporting information


**Appendix S1** England clinical guidelines relevant to EIP services.

**Appendix S2** Ethical requirements of service delivery: Sample quotes.

**Appendix S3** Moral attributes of clinicians: Sample quotes.

## Notes

1 In April 2016, NHS England, NICE and the National Collaborating Centre for Mental Health published the document: “Implementing the Early Intervention in Psychosis Access and Waiting Time Standard: Guidance” (2016). This document was published after our analysis had been concluded and is not included in this review.

2 Among NICE pathways originally identified as addressing the clinical management of psychosis and schizophrenia (*n* = 9), six documents were deemed relevant for the present analysis, as they address principles of care, treatment and recovery of (a) children and young people with psychosis and schizophrenia; and (b) prevention in adults at risk of developing psychosis.
