# Supplementary material for: What constitutes ‘good practice’ in early intervention for psychosis? Analysis of clinical guidelines
Source: Child Adolesc Ment Health. 2017 Aug 8;23(3):185–93. doi: 10.1111/camh.12229 (PMC6120554; doi:10.1111/camh.12229)
Supplement: Supplementary file 1 — Appendix S1 England clinical guidelines relevant to EIP services. [file CAMH-23-185-s001.docx]

**Appendix S1. England Clinical Guidelines relevant to EIP services**

1. The IRIS guideline (IRIS) is the key document specifically designed to provide good practice guidance for EIP services in England.
2. NICE guidelines, pathways and quality standards included in the analysis refer to EIP services in different ways:
   - CG155 provides guidance for the management of children and young people with psychosis and schizophrenia up to the age of 18, covering CAMHS and EIP services and placing great emphasis on the management of the prodromal period (see p. 4-5)
   - CG178 covers the management of psychosis and schizophrenia in adults, focusing on the importance of early intervention (p. 5), prevention (p. 8), and describing structure and aims of EIP services in England (p. 16)
   - CG120 covers the management of psychosis with substance misuse in over 14s referred to CAMHS and EIP services
   - QS102 represents the quality standards provided by NICE for the organization of CAMHS and EIP services dealing with children and young people under 18 with bipolar disorder, psychosis and schizophrenia, and it underlines the importance of providing intervention during the prodromal period (p. 6)

**Supporting Online Information for** ***What constitutes ‘good practice’ in early intervention for psychosis? Analysis of clinical guidelines* by Singh et al.**
