# Supplementary material for: What constitutes ‘good practice’ in early intervention for psychosis? Analysis of clinical guidelines
Source: Child Adolesc Ment Health. 2017 Aug 8;23(3):185–93. doi: 10.1111/camh.12229 (PMC6120554; doi:10.1111/camh.12229)
Supplement: Supplementary file 2 — Appendix S2 Ethical requirements of service delivery: Sample quotes. [file CAMH-23-185-s002.docx]

| **Appendix S2.** Ethical Requirements of Service Delivery: Sample Quotes | | | | |
| --- | --- | --- | --- | --- |
|  | **Theme** | **Sample Quote** | **Doc** | **Page** |
| **Service Structure** | Collaboration with other services | *Mental health services should work in partnership with local stakeholders, including those representing black, Asian and minority ethnic groups, to enable people with mental health problems, including psychosis or schizophrenia, to stay in work or education and to access new employment (including self- employment), volunteering and educational opportunities.* | CG178 | 32 |
|  | Promote creative expression | *Arts therapies should combine psychotherapeutic techniques with activity aimed at promoting creative expression, which is often unstructured and led by the service user. Aims of arts therapies should include: enabling people with psychosis or schizophrenia to experience themselves differently and to develop new ways of relating to others; helping people to express themselves and to organise their experience into a satisfying aesthetic form; helping people to accept and understand feelings that may have emerged during the creative process (including, in some cases, how they came to have these feelings) at a pace suited to the person.* | CG178 | 25 |
|  | Timely intervention | *[…] the principle is the same: to intervene as early and effectively as possible to prevent or limit the secondary and tertiary consequences of these disorders […]* | IRIS | 5 |
|  | Raise awareness | *Raise awareness about how primary care can use its skills and capability to improve the physical care pathways of those with early psychosis.* | PCG | 2 |
|  | Focus on recovery | *Intervening early and effectively in the course of psychosis can limit initial problems and improve long-term prospects for recovery.* | IRIS | 12 |
| **Codes of Ethics** | Informed consent | *Health and social care professionals should ensure that they: can assess capacity and competence, including 'Gillick competence', in children and young people of all ages […]* | CG155 | 12 |
|  | Privacy | *When working with children and young people with psychosis or schizophrenia and their parents or carers: make sure that discussions take place in settings in which confidentiality, privacy and dignity are respected.* | CG155 | 13 |
|  | Confidentiality | *[…] be clear with the child or young person and their parents or carers about limits of confidentiality (that is, which health and social care professionals have access to information about their diagnosis and its treatment and in what circumstances this may be shared with others).* | CG155 | 13 |
| **Inclusive** | Equal access | *All children and young people with psychosis or schizophrenia have equal access to services based on clinical need and irrespective of gender, sexual orientation, socioeconomic status, age, background (including cultural, ethnic and religious background) and any disability.* | CG155 | 16 |
|  | Eliminate discrimination | *Services that are sensitive to the needs of local populations and the diversity within them seeking to eliminate discrimination […]* | ABA | 11 |
|  | Reduce stigma | *Offer low stigma approach to encourage help seeking […]* | IRIS | 15 |
| **Patient & Family Centred** | Needs and preferences | *Treatment and care should take into account individual needs and preferences.* | CG178 | 7 |
|  | Tailored services | *Service resources and required staff skill-sets will need to be tailored to the needs of clients at different stages of life-development.* | IRIS | 5 |
|  | Good communication | *Good communication between health and social care practitioners and children and young people with bipolar disorder, psychosis or schizophrenia, and their parents or carers (if appropriate), is essential.* | QS102 | 47 |
|  | Improve quality of life | *An essential skill of EIP practitioners includes the ability to support service users to generate and mobilise social resources to build their social capital as a way to enhance quality of life and improve recovery.* | IRIS | 24 |
| **Appropriate Treatment** | Responsibility | *The secondary care team should maintain responsibility for monitoring service users' physical health and the effects of antipsychotic medication […]* | CG178 | 20 |
|  | Shared decision-making | *The choice of antipsychotic medication should be made by the service user and healthcare professional together, taking into account the views of the carer if the service user agrees. Provide information and discuss the likely benefits and possible side effects of each drug […]* | CG178 | 8 |
|  | Avoid over diagnosis | *Treatment needs to focus on management of symptoms and sufficient time needs to be allowed for symptoms to stabilise before a diagnosis is made.* | MHPIG | 45 |
|  | Cost-effectiveness | *[…] it is important to emphasise that Early Intervention in Psychosis does not describe an intervention but rather a distinctive model of service and ethos/philosophy of care, with an evidence base of clinical and cost-effectiveness and positive service user evaluation.* | IRIS | 4 |
