# Supplementary material for: What constitutes ‘good practice’ in early intervention for psychosis? Analysis of clinical guidelines
Source: Child Adolesc Ment Health. 2017 Aug 8;23(3):185–93. doi: 10.1111/camh.12229 (PMC6120554; doi:10.1111/camh.12229)
Supplement: Supplementary file 3 — Appendix S3 Moral attributes of clinicians: Sample quotes. [file CAMH-23-185-s003.docx]

| **Appendix 3S.** Moral Attributes of Clinicians: Sample Quotes | | | | |
| --- | --- | --- | --- | --- |
|  | **Theme** | **Sample Quote** | **Doc** | **Page** |
| **Competency** | Appropriate training | *Training programmes and written guidance for GPs and other key agencies are needed on the importance of early detection and how to refer people with potential early psychosis.* | MHPIG | 46 |
|  | Awareness | *[…] be aware of possible variations in the presentation of mental health problems in children and young people of different genders, ages, cultural, ethnic, religious or other diverse backgrounds* | CG155 | 15 |
|  | Being cautious | *Be particularly cautious when considering high-potency antipsychotic medication (such as haloperidol) in children and young people, especially those who have not taken antipsychotic medication before, because of the increased risk of acute dystonic reactions in that age group.* | CG155 | 32 |
|  | Responsibility | *[…] the guidance does not override the individual responsibility of healthcare professionals to make decisions appropriate to the circumstances of the individual patient, in consultation with the patient and/or guardian or carer, and informed by the summaries of product characteristics of any drugs.* | CG178 | 57 |
|  | Appropriate supervision | *Health and social care professionals inexperienced in working with children and young people with psychosis or schizophrenia from diverse ethnic and cultural backgrounds, and their parents or carers, should seek advice and supervision from healthcare professionals who are experienced in working transculturally.* | NP1 | 7 |
| **Empathy** | Provide support (to users and families) | *Take time to build supportive and empathic relationships as an essential part of care.* | CG178 | 10 |
|  | Foster autonomy | *When working with children and young people with psychosis or schizophrenia: aim to foster autonomy, promote active participation in treatment decisions, and support self-management and access to peer support in children and young people of an appropriate developmental level, emotional maturity and cognitive capacity.* | CG155 | 13 |
|  | Give hope / being positive | *Provide treatment and care in the least restrictive and stigmatising environment possible and in an atmosphere of hope and optimism […]* | CG178 | 27 |
| **Sensitivity** | Being respectful | *Be respectful of and sensitive to children and young people's gender, sexual orientation, socioeconomic status, age, background (including cultural, ethnic and religious background) and any disability.* | CG155 | 15 |
|  | Tailor communication | *When communicating with children and young people with psychosis or schizophrenia and their parents or carers: take into account the child or young person's developmental level, emotional maturity and cognitive capacity including any learning disabilities, sight or hearing problems or delays in language development; use plain language where possible and clearly explain any clinical language; check that the child or young person and their parents or carers understand what is being said; use communication aids (such as pictures, symbols, large print, braille, different languages or sign language) if needed.* | CG155 | 14 |
| **Trustworthiness** | Promote engagement | *Early engagement is the most important initial therapeutic goal.* | IRIS | 17 |
|  | Build trust | *Take time to build trusting, supportive, empathic and non-judgemental relationships as an essential part of care.* | CG155 | 13 |
|  | Foster good communication | *When carrying out an assessment:*  *Ensure there is enough time for: the child or young person and their parents or carers to describe and discuss their problems; summarising the conclusions of the assessment and for discussion, with questions and answers*  *Explain and give written material in an accessible format about any diagnosis given; give information about different treatment options, including pharmacological and psychological interventions, and their benefits and side effects, to promote discussion and shared understanding; offer support after the assessment, particularly if sensitive issues, such as childhood trauma, have been discussed* | CG155 | 19 |
|  | Account for interdependence | *Foster a collaborative approach that supports both service users and carers, and respects their individual needs and interdependence.* | CG178 | 13 |
